# Child Expelled from the Womb after Interment

**Published:** 1839-01-19

**Authors:** 


*Child expelled from the Womb after Interment.*—A well authenticated account of this curious occurrence is reported in the November number of the Edinburgh Medical and Surgical Journal. The mother had died in labour, after forty eight-hours of severe, but ineffectual pains. The cervix uteri was diseased, and the os uteri had never become sufficiently dilated to justify artificial delivery. Three weeks after burial, the body was disinterred, in consequence of reports that she had not been properly treated. "When the shroud, which covered her, was removed, a child of near eight months growth was lying on the thighs, the head downwards, and one foot and the funis still connected in the vagina." The inference is, that the child was expelled in the grave. This, it is suggested, might have been "owing to the resistance of the *os uteri* and *cervix* being overcome, 1*st*, by the relaxation occasioned by the act of dissolution, and 2*d*, by the collapse of the cauliflower excrescences, which seem to have been rigid and swollen during life, but were totally gone on the necroscopic inspection; and *secondly*, by the distension of the parietes of the abdomen from the gas evolved during decomposition, the *os* and *cervix* reacting upon the most yielding parts, which were now the *os* and *cervix uteri*, and thus, by their common elasticity only, expelling the child. The collapsed and flaccid state of the *uterus*, the very relaxed *cervix* and mouth, the distended abdomen, all concur in supporting this view."

Another explanation offered, is, that "during the changes which take place after death, a quantity of gas would be excreted into the internal cavities of the body. The effect of this would very likely be to drive out the child, upon the same principle as a cork is driven from a bottle of ginger-beer or soda water."

